# The Institutional Library

**Published:** 1914-06-20

**Authors:** 


### THE INSTITUTIONAL LIBRARY.

### Scientific By-paths.

Exercises for Women. By Florence Bolton. (Funk and Wagnalls Co. 1914. Pp. 141. Illustrated. Price 4s. net.)

This book is, on the whole, sensible and reasonable, as well as readable. The first part, nearly one-half of the whole book, contains an exposition of various hygienic principles, observance of which is highly necessary for civilised beings unable to get the outdoor exertion of their primitive ancestors. This sort of instruction is so often either inaccurate or pedantic that it is a pleasure to read a book in which it is neither one nor the other, but rational and practical. Then follow chapters dealing with simple exercises, especially adapted for the use of women. These, taken in bulk, are equally worthy of praise. The book should certainly have much popularity with those for whom it is written.

A Text-book of General Embryology. By Professor William E. Kellicott. (London: Constable and Co. 1914. Pp. 376. Price 10s. 6d. net.)

This monograph on one of the most fascinating of those scientific bypaths down which the medical student is often tempted to stray is elementary in scope and deals with general principles rather than with concrete examples of them. It is not arranged on the "type" system so dear to British authors, but is perhaps all the better in consequence. The teaching is sound, the arrangement perspicuous, the exposition lucid, the type most excellent, and the bibliographies appended to each chapter comprehensive. In a word, it is a volume we can thoroughly recommend as an introduction to the study of the science with which it deals.

### The Practising Operator.

Manual of Surgical Treatment. By Sir W. W. Cheyne, Bart., C.B., F.R.C.S., F.R.S., and F. F. Burghard, M.S., F.R.C.S. New Edition. Edited by T. P. Legg, M.S., F.R.C.S., and Arthur Edmunds, M.S., F.R.C.S. Vol. V. (London: Longmans, Green and Co. 1913. Pp. 619. Price 2s. net.)

The fifth and last volume of the new edition of this famous manual is, if anything, of greater interest to the practising operator, whether he be a general surgeon or a general practitioner, than any of the four preceding parts, for it includes the surgical affections of the pancreas, liver, spleen, neck, breast, thorax, and genito-urinary system; in other words, a number of the organs whose derangements most frequently require surgical intervention for their relief. Moreover, the regions in question are pre-eminently those in which surgery has taken vast strides during the past fifteen years; and it is gratifying to note that in nearly every respect this volume is well up to date and incorporates those views which are being held by the most trustworthy men of the modern surgical school. In the surgery of the kidneys, bladder, and accessory organs the revolution in surgical teaching since the original edition of this manual has been particularly startling; and one notes with pleasure that the editors have assimilated these advances, and that, with the help of Mr. J. W. Thomson Walker, they reflect them faithfully in the chapters dealing with the diseases in question. Some of the illustrations are distinctly crude, and do not compare favourably with the artistic products which characterise the best class of American text-books; but for practical purposes they are for the most part quite sufficient to bring out the points discussed in the text. Nos. 43 and 98 seem to be exceptions to this statement; neither of them is of help, and both should be replaced or omitted. In general, the volume will give unqualified satisfaction to British surgeons, and is an unequivocal credit to British surgery. The series is now complete, and forms a really superb text-book of surgical treatment, for which the authors and editors deserve unstinted praise and congratulations.

A Complete Handbook of Midwifery for Midwives and Nurses. By J. K. Watson, M.D. (The Scientific Press. Third Edition. Thoroughly Revised. Pp. 360. Price 6s. net.)

This text-book has attained a thoroughly well-deserved popularity among nurses and midwives, as testified by the exhaustion of the two previous editions. As the author truly stated in his preface to the original edition, his aim was to include too much rather than too little, and time has proved the wisdom of his policy. It may still be true that there is a good deal included in Dr. Watson's text-book which the practising midwife or maternity nurse does not need to know; but the educational evolution of the midwife has proceeded steadily in recent years, and there is very little in the book which we should care to see omitted. The teaching is sound and sensible; and we have every confidence that the success of this revised edition will be as marked as that of the previous issues. There is no better book of its size and scope on the market.

